# The Theory of Planned Behavior to Predict Protective Behavioral Intentions against PM2.5 in Parents of Young Children from Urban and Rural Beijing, China

**DOI:** 10.3390/ijerph15102215

**Published:** 2018-10-10

**Authors:** Shumei Liu, Yi-Te Chiang, Chie-Chien Tseng, Eric Ng, Gwo-Liang Yeh, Wei-Ta Fang

**Affiliations:** 1 Department of Health Promotion and Health Education, National Taiwan Normal University, Taipei 106, Taiwan; shumei80405010e@gmail.com (S.L.); t09008@ntnu.edu.tw (G.-L.Y.); 2 Graduate Institute of Environmental Education, National Taiwan Normal University, Taipei 116, Taiwan; faratajiang@gmail.com; 3 School of Management and Enterprise, University of Southern Queensland, Toowoomba, QLD 4350, Australia; eric.ng@usq.edu.au

**Keywords:** theory of planned behavior, anti-PM2.5 behavioral intention, young children’s parents, rural and urban areas

## Abstract

Smog and air pollution have fast become significant environmental problems and are attributed to rapid global industrialization and urbanization. Emissions of fine particulate matter with an aerodynamic diameter of ≤2.5 μm (PM2.5) increase smog and air pollution, with strong impacts on human health. Children are particularly vulnerable. While increasing studies are being conducted on the behaviors leading to PM2.5 toxicity from the perspective of environmental toxicants, there is a lack of research on factors influencing anti-PM2.5 behavioral intentions. Thus, this study aims to narrow this gap by adapting the theory of planned behavior framework to investigate the effects of attitude, subjective norms, and perceived behavioral control on protective behavioral intentions against PM2.5. In total, 1277 online questionnaires were collected from parents of young children living in urban and rural areas of Beijing, and the data was analyzed using correlation, regression, and path analyses. Results revealed that there were significant differences between parents from urban and rural areas in terms of attitude (*t* = 4.727 > 1.96, *p* < 0.001), subjective norms (*t* = 5.529 > 1.96, *p* < 0.001), perceived behavioral control (*t* = 6.155 > 1.96, *p* < 0.001), and anti-PM2.5 behavioral intentions (*t* = 6.838 > 1.96, *p* < 0.001). Path analysis revealed that parents from urban and rural areas had different behavioral intention paths. For urban parents, the findings indicated that subjective norms (β = 0.73, *t* = 21.84 > 3.29) and perceived behavioral control (γ = 0.22, *t* = 6.12 > 3.29) had direct impacts on anti-PM2.5 behavioral intentions. In contrast, the attitudes (γ = 0.39, *t* = 3.74 > 3.29) and subjective norms (β = 0.60, *t* = 8.55 > 3.29) of rural parents were found to directly influence anti-PM2.5 behavioral intentions.

## 1. Introduction

Rapid global industrialization and urbanization have led to increasing environmental problems, attracting widespread attention. Among other issues, smog and air pollution are compelling environmental problems with significant negative impacts on human health, the national economy, and medical expenditure [1,2,3,4]. Fine particulate matter with aerodynamic diameter of ≤2.5 μm (PM2.5) is a main source of smog [5,6] that penetrates and settles deep into the alveoli and can result in damage to the respiratory system [7]. Empirical studies have shown that long-term exposure to PM2.5 can lead to cardiovascular and respiratory diseases and that the daily numbers of deaths, stroke events, and hospital and emergency room visits are positively associated with increasing PM2.5 concentrations [8,9]. Therefore, reducing the concentrations of PM2.5 will not only help reduce unnecessary medical expenses and prevent further mortality but also improve the economy [10].

Air pollution is harmful to one’s health well-being, and children in general are more sensitive to PM2.5 pollution, with infants and toddlers being particularly vulnerable [11,12,13,14]. A case series reported that airborne contaminants and particles, which are harmful to children’s respiratory systems, significantly increased the hospitalization rates of children with respiratory diseases [15]. Short-term exposure to air pollution can lead to hospitalization of children with asthma, and younger children are considered to be more vulnerable [12]. In addition to outdoor air pollution, indoor air pollution in downtown areas of large cities can also increase the asthma risk in children [16]. Many prior studies have focused on older children but rarely on younger children [17,18]. Parents are important figures in the lives of their children, and are key players in shaping their behaviors. Thus, parents’ attitudes and behaviors toward the environment can influence those of their children. One study suggested that parents’ perceptions and behavior at home with respect to smoking affected the exposure of their preschool children to air pollution, as demonstrated by altered urine composition [19]. Hence, parents’ attitudes toward exposure to environmental chemicals can potentially be associated with chemical concentrations in their children’s urine [20]. On the other hand, some studies have argued that children, through their learning about the environment from school education, can also transmit environmental knowledge and attitudes to their parents, thus affecting their parents’ environmental behaviors [21,22].

According to the World Health Organization, diseases caused by environmental pollution account for 21% of the total disease burden in China [23]. The capital city of China, Beijing, was one of the most PM2.5 polluted cities in the world from 2014 to 2016, leading to significant economic losses and health threats [24]. Since then, reduction in and protection against PM2.5 has become a prime focus for the government and public at large [25]. As the capital city of one of the world’s largest economies, Beijing has numerous highly developed economic and trade centers, as well as abundant natural resources spread across its urban and rural areas. Urban and rural areas in Beijing have different PM2.5 emission sources; for instance, industrial emissions (e.g., metallurgy waste and sulfates) are more evident in rural areas, whereas anthropogenic emissions (e.g., transportation and coal burning waste) are more noticeable in urban areas [26]. One study revealed that the mortality rate as a result of air pollution in Beijing was higher in urban areas than in rural areas [1].

Several studies have explored PM2.5 in relation to reduction, control, and protection technologies, such as PM2.5 source analysis [27], chemical composition analysis [6], and technological pollution control [28]. While other studies have also explored PM2.5 reduction behaviors from a household perspective [29,30], the protective behavioral intentions against PM2.5 (hereafter, anti-PM2.5 behavioral intentions) from the health management and human behavior perspectives have seldom been investigated. In addition to PM2.5 source reduction and control, methods for cultivating anti-PM2.5 behavioral intentions with respect to health hazards in families and societies are also crucial for resolving PM2.5- and smog-related environmental problems. Therefore, further insights into understanding the impact path of anti-PM2.5 behavioral intentions are essential.

The present study aims to analyze the behavioral structure of parents of young children against PM2.5-related health hazards by using the key concepts of the theory of planned behavior (TPB) and seeks to explain the relationship between psychosocial factors and anti-PM2.5 behavioral intentions. Ajzen [31] developed TPB by extending the concepts of rational behavior and perceived behavioral control (PBC). The TPB has since been widely applied in the research area of health management behaviors, such as sexual behavior [32,33], smoking [34,35], alcoholism [36], and drug abuse [37]. In addition, anti-PM2.5 behavioral intentions comprise not only the health protection behaviors but also the environmental-friendly behaviors of individuals [29]. TPB is currently one of the most commonly used theories in research studies on the interpretation and prediction of various environmental-friendly behaviors [38,39]. Accordingly, there are three main concepts in TPB that influence behavioral intentions: (1) attitude (which refers to the degree to which an individual supports a particular behavior [31]); (2) PBC (which refers to the ability of an individual to control the external environment [40]); and (3) subjective norms (which refer to an individual’s perceptions of external social pressures [31]). Together, these factors influence the behavioral intentions and can directly predict the behavioral factors of individuals’ actions [31,40].

Given the above, this research study seeks to investigate the influence of attitude, PBC, and subjective norms on the anti-PM2.5 behavioral intentions of young children’s parents in Beijing. Specifically, this study will explore the differences in factors influencing the anti-PM2.5 behavioral intentions of urban and rural parents residing in Beijing. Furthermore, the path structure model will be used to mediate the intervening variables by using subjective norms and to determine the impact of the attitude and PBC of two groups of parents on their anti-PM2.5 behavioral intentions.

## 2. Materials and Methods

### 2.1. Research Hypotheses

Based on the extant literature discussed earlier, the following hypotheses are proposed.

**Hypothesis 1** **(H1)**. *The attitude of urban parents positively influences their anti-PM2.5 behavioral intentions [29,41,42,43].*

**Hypothesis 2** **(H2)**. *The attitude of urban parents positively influences their subjective norms. A significant correlation exists between subjective norms and attitude [44,45,46]. Attitude directly affects subjective norms [45,47], whereas subjective norms directly affect attitude [48,49,50,51,52,53]; thus, attitude is regarded as an intervening variable between subjective norms and behavioral intentions.*

**Hypothesis 3** **(H3)**. *The PBC of urban parents positively influences their anti-PM2.5 behavioral intentions [29,43,54].*

**Hypothesis 4** **(H4)**. *The PBC of urban parents positively influences their subjective norms. A significant correlation exists between subjective norms and PBC [44,45,46]. Subjective norms directly affect PBC and indirectly affect behavioral intentions [43], whereas subjective norms and PBC interact with behavioral intentions [53,55]. However, whether PBC has positive, direct influence on subjective norms remains unclear and needs further exploration.*

**Hypothesis 5** **(H5)**. *The subjective norms of urban parents positively influence their anti-PM2.5 behavioral intentions [30,41,42].*

**Hypothesis 6** **(H6)**. *The attitude of rural parents positively influences their anti-PM2.5 behavioral intentions [29,41,42,43].*

**Hypothesis 7** **(H7)**. *The attitude of rural parents positively influences their subjective norms. A significant correlation exists between subjective norms and attitude [44,45,46]. Attitude directly affects subjective norms [45,47], whereas attitude is an intervening variable between subjective norms and behavioral intentions [48,49,51,52,53].*

**Hypothesis 8** **(H8)**. *The PBC of rural parents positively influences their anti-PM2.5 behavioral intentions [29,43,54].*

**Hypothesis 9** **(H9)**. *The PBC of rural parents positively influences their subjective norms. A significant correlation exists between subjective norms and PBC [44,45,46]. Subjective norms directly affect PBC and indirectly affect behavioral intentions [43], whereas subjective norms and PBC interact with behavioral intentions [53,55].*

**Hypothesis 10** **(H10)**. *The subjective norms of rural parents positively influence their anti-PM2.5 behavioral intentions [30,41,42].*

### 2.2. Research Area

The study was conducted in Beijing, the capital city of People’s Republic of China, which is situated in the northern part of the country. Beijing is the second largest city in China and is made up of 16 administrative districts. It is a political, economic, and cultural center which covers an area of 16,808 km^2^. Beijing has a population of around 21.71 million and is considered to have an extremely high resident density, with 1289 people per square kilometer. The city is divided into metropolitan (with highly developed economic and trade centers) and rural areas, with a population ratio of 86.5:13.5 as per the Beijing Municipal Bureau of Statistics (2017) [56]. Major industries in Beijing include tourism, telecommunications equipment, electronics, chemicals, transportation equipment, machinery, textiles, metallurgy, garments, and household appliances. Beijing’s industrial production is dominated by heavy industries, which contributed to 84.9% of Beijing’s gross industrial output in 2016.

In 2013, China experienced severe smog pollution, wherein 74 cities were covered with heavy smog for 146 days. Among these cities, the Beijing–Tianjin–Hebei region was reported to have the most severe air pollution. Beijing, where the pollution persisted for 190 days [57], was regarded as one of the most severely smog-polluted areas by the Chinese government [58]. During 2017, the air quality in Beijing was very poor, and PM2.5 was the primary air pollutant. The average annual concentration of PM2.5 in various administrative regions of Beijing varied from 49 to 67 μg/m^3^, failing to meet national standards. According to Beijing’s Environmental Protection Bureau, PM2.5 was mainly associated with traffic (45%), dust (14%), industry (12%), daily life activity (12%), coal-fired activity (4%), and other sources (12%) [56]. Recent statistics revealed that Beijing has 445,535 children enrolled in kindergartens for the academic year 2017–2018. A survey was conducted in April 2018, when Beijing was experiencing severe smog pollution, to understand the views of the parents of young children from urban and rural areas of Beijing with respect to protection against PM2.5.

### 2.3. Participants

**Figure 1:**
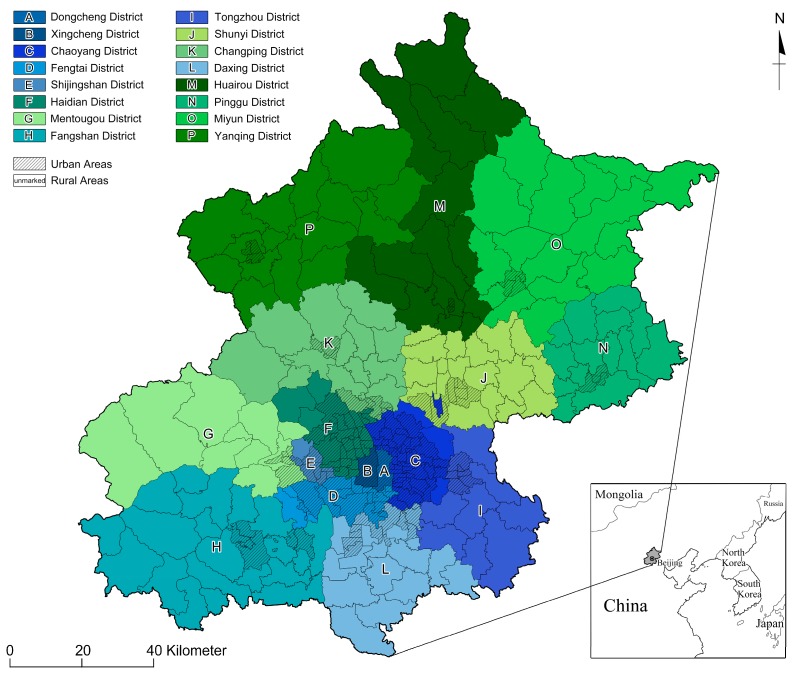
A map showing the 16 stratified districts in Beijing where this study took place.

This study used a stratified sampling method with an online questionnaire survey to identify participants from 16 districts (as shown in Figure 1) in Beijing. We contacted the teachers and parents of children in kindergartens in various districts of Beijing with an equal ratio from urban and rural areas. We then joined the kindergarten parents’ groups on WeChat, which is a social networking application commonly used by Chinese residents. We sent electronic questionnaire with the associated URL using Wen juan xing (WJX) technology to these groups, inviting parents to participate. This study involved no invasive measures. We issued consent forms to parents, class teachers, and kindergarten directors to seek their agreement to participate in this study, at the same time informing them of their rights to withdraw at any time in this anonymous questionnaire survey.

According to the National Taiwan Normal University Research Ethics Committee, our research does not fall within the scope of the Human Subjects Research Act. Therefore, the committee approved the study protocol (201804HS008) and agreed on active informed consent being obtained from class teachers and kindergarten directors, with parents having the option to opt out of the study. A total of 3000 questionnaires were distributed on the WeChat groups, of which 1300 responses were received. However, 23 invalid questionnaires were eliminated, and as such the remaining 1277 questionnaires were analyzed.

### 2.4. Measures

This study was based on a substantial body of TPB literature and sought to measure three key TPB dimensions, namely attitude, PBC, and subjective norms, which were considered to influence the anti-PM2.5 behavioral intentions. The questions in the questionnaire were mainly adapted from previous research on environmental behaviors, specifically related to the scales for attitude [59], subjective norms [17], and environmental behaviors [59]. To determine the appropriateness and comprehensibility of the questions, six experts from the health promotion, health education, or environmental education domains reviewed the content validity of the questionnaire by administering pretests to 30 parents of young children in Beijing. Consequently, some minor changes were made to the questionnaire and were subsequently used for the actual survey. These subtle changes, without changing the original meaning of the aforementioned research questions, were aimed at simplifying wording by including current plain language and clearer terms in the local context so that the parents could understand the questions accurately.

The Statistical Package for Social Sciences (version 23) was used for analyzing descriptive demographic statistics and items to calculate the total number of occurrences, percentages, means, and standard deviations (SDs). One-way analysis of variance was used to determine region-, gender-, education-, and income-related differences in key dimensions, namely attitude, subjective norms, PBC, and anti-PM2.5 behavioral intentions. Pearson’s correlation coefficient was used to measure the strength and direction of the relationship between these key dimensions. This study has adopted a five-point Likert scale (i.e., with 1 representing “Strongly disagree” through to 5 representing “Strongly agree”) as the form of measurement.

## 3. Results

### 3.1. Descriptive Statistics

Of the 1277 valid questionnaires returned, there were 1012 (79.2%) received from participants from urban areas, with the remaining 265 (20.8%) from participants from rural areas in Beijing. The overall findings indicated that females (69.4%) were better represented than males (30.6%) in this study. In urban areas, 70.8% of respondents were female and 29.2% were male. In rural areas, 64.4% of respondents were female and 35.6% were male. Results revealed significant differences in attitude (degree of freedom, df = 1275, two-tailed *t*-test, *t* = 4.727 > 1.96, *p* < 0.001), subjective norms (df = 1275, two-tailed, *t* = 5.529 > 1.96, *p* < 0.001), PBC (df = 1275, two-tailed, *t* = 6.155 > 1.96, *p* < 0.001), and anti-PM2.5 behavioral intentions (df = 1275, two-tailed, *t* = 6.838 > 1.96, *p* < 0.001), where urban parents’ responses scored considerably higher than those of the rural parents. There were also significant differences between females and males in relation to their attitude (df = 1264, two-tailed, *t* = 4.113 > 1.96, *p* < 0.001), subjective norms (df = 1264, two-tailed, *t* = 3.293 > 1.96, *p* = 0.001), PBC (df = 1264, two-tailed, *t* = 4.088 > 1.96, *p* < 0.001), and anti-PM2.5 behavioral intentions (df = 1264, two-tailed, *t* = 4.874 > 1.96, *p* < 0.001). Within the urban areas, responses from the females scored significantly higher in attitude (df = 1000, two-tailed, *t* = 3.422 > 1.96, *p* < 0.001), subjective norms (df = 1000, two-tailed, *t* = 2.268 > 1.96, *p* = 0.024), PBC (df = 1000, two-tailed, *t* = 3.257 > 1.96, *p* = 0.001), and anti-PM2.5 behavioral intentions (df = 1000, two-tailed, *t* = 3.216 > 1.96, *p* < 0.001). Similarly, in the rural areas, the responses in subjective norms (df = 262, two-tailed, *t* = 2.075 > 1.96, *p* = 0.039), PBC (df = 262, two-tailed, *t* = 2.162 > 1.96, *p* = 0.032), and anti-PM2.5 behavioral intentions (df = 262, two-tailed, *t* = 2.627 > 1.96, *p* = 0.009) scored significantly higher in females than in males. Table 1 below briefly outlines selected demographic findings in terms of attitude, subjective norms, PBC, and behavioral intentions.

As shown in Table 2, the overall education background for all parents was categorized into high school graduates and below (13.55%), university or college graduates (72.04%), and university postgraduates (14.41%). Findings indicated that the majority of parents in urban areas were university or college graduates (73.12%), followed by university postgraduates (16.21%), and then high school graduates or below (10.67%). As for parents in the rural areas, 67.92% were university or college graduates, 24.53% were high school graduates or below, and 7.55% were university postgraduates. Results (see Table 3) also revealed that parents from urban areas had received significantly higher levels of education than those from rural areas (df = 1275, two-tailed, *t* = 8.273 > 1.96, *p* < 0.001).

In terms of income levels (as shown in Table 4), the overall results for all parents were classified as follows: less than RenMinBi, Chinese Yuan, RMB 100,000 (29.37%), RMB 110,000–200,000 (35.08%), RMB 210,000–300,000 (22.40%), RMB 310,000–400,000 (7.44%), and over RMB 410,000 (5.72%). Findings suggested that urban parents with annual incomes of RMB 110,000–200,000 accounted for 36.36% of respondents, followed by those with annual incomes of less than RMB 100,000 (26.48%), RMB 210,000–300,000 (22.92%), RMB 310,000–400,000 (7.61%), and over RMB 410,000 (6.62%). For rural parents, 40.38% had annual incomes of less than RMB 100,000, with those remaining having incomes of RMB 110,000–200,000 (30.19%), RMB 210,000–300,000 (20.38%), RMB 310,000–400,000 (6.79%), and over RMB 410,000 (2.26%). As presented in Table 5, findings indicated that the annual incomes of urban parents were significantly higher than those of rural parents (df = 1275, two-tailed, *t* = 4.032 > 1.96, *p* < 0.001).

Findings for the attitude-related items are presented in Table 6. The overall attitude mean score was 4.16. Among the four attitude-related items, “I care about whether smog affects the health of the people” and “I care about some problems of domestic industrial pollution” had the highest mean scores. This was followed by “Smog pollution is the result of human activities destroying the environment”, and “I care about the environmental problems resulting from economic development.” The results indicated an internal consistency reliability measurement with the Cronbach’s α value of 0.960 for the attitude-related items.

Results (see Table 7) indicated that the mean score for the overall subjective norms was 4.17. There were four subjective norms-related items: “People important to me hope that I wear a surgical mask and get my children to wear surgical masks when there is smog”, and “People important to me hope that I choose environment-friendly products” received the highest and lowest mean scores, respectively. Other items included: “People important to me hope that I walk, ride a bicycle or use modes of public transport”, and “People important to me hope that I participate in environmental activities to improve smog pollution,”. The Cronbach’s α value of 0.948 suggested internal consistency for the subjective norm-related items.

Findings showed that the mean score for overall PBC was 4.22. There were five items related to PBC, namely, “I can install air purification equipment with good purification and ventilation functions”, “I pay attention to poor air quality and respond by taking measures”, “I can give environmental education to my children before they start school (in the kindergarten stage)”, “We can carry out protective education and work against PM2.5 at kindergartens”, and “I can protect my children against PM2.5”. Table 8 presents the respective mean scores. There was a consistent reliable measurement for the PBC-related items with the Cronbach’s α value of 0.971.

Findings (see Table 9) revealed a mean score of 4.22 for the overall behavioral intentions. There were six items about anti-PM2.5 behavioral intentions, of which “When the smog reaches the warning level, I get the children to stay indoors” had the highest mean score. This was followed by “When the smog is serious, I will get the children to wear surgical masks fitting their faces tightly”, “I pay attention to the Air Quality Index (AQI) every day to remind the children to pay attention to protecting themselves against smog”, “I advise others against polluting the environment”, “I participate in activities related to environmental protection”, and “I use fuel that has less impact on the environment even if it costs much more”. The internal consistency reliability for the anti-PM2.5 behavioral intention-related items was measured with the Cronbach’s α value of 0.960.

### 3.2. Correlation Analysis

According to the correlation analysis (see Table 10), the relationship between subjective norms and anti-PM2.5 behavioral intentions (*r* = 0.888, *p* < 0.001) was the strongest. Findings also revealed high correlations between: (1) attitude and subjective norms (*r* = 0.739, *p* < 0.001); (2) attitude and PBC (*r* = 0.844, *p* < 0.001); (3) attitude and anti-PM2.5 behavioral intentions *(r* = 0.751, *p* < 0.001); (4) subjective norms and PBC (*r* = 0.804, *p* < 0.001); and (5) PBC and anti-PM2.5 behavioral intentions (*r* = 0.815, *p* < 0.001). Thus, all four dimensions in this study were significantly correlated and had existing inter-relationships in their paths towards anti-PM2.5 behavioral intentions.

### 3.3. Regression and Path Analysis

**Figure 2:**
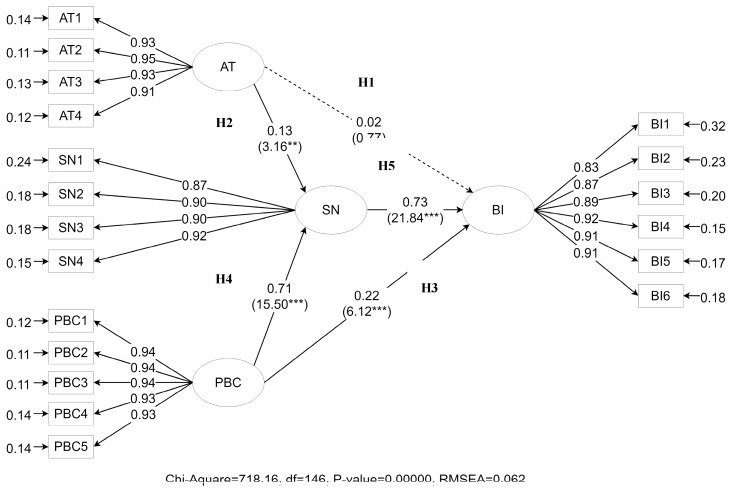
Path diagram of attitude (AT), perceived behavioral control (PBC), subjective norms (SN), and anti-PM2.5 behavioral intentions (BI) of parents from the urban areas in Beijing.

Path analysis performed on Lisrel 9.2, (Scientific Software International, Skokie, IL, USA) revealed that parents from urban and rural areas had different behavioral intention paths. For urban parents, the model goodness of fit index (GFI), comparative fit index (CFI), and non-normed fit index (NNFI) values were 0.923, 0.978, and 0.975, respectively (all > 0.90: good fit). The root-mean-square error of approximation (RMSEA) was 0.062 (<0.080: acceptable fit), and the normed chi-squared value was 718.16 (>3). According to the model structure of anti-PM2.5 behavioral intentions as shown in Figure 2, PBC had a direct impact on their anti-PM2.5 behavioral intentions (H3 was supported), whereas attitude had no direct influence (H1 was rejected), but they could be developed through subjective norms. The path analysis results also indicated that anti-PM2.5 behavioral intentions were influenced by their subjective norms (H5 was supported), which in turn were significantly influenced by attitude (H2 was supported) and PBC (H4 was supported). Thus, the influence of PBC was the strongest, whereas attitude was the weakest.

**Figure 3:**
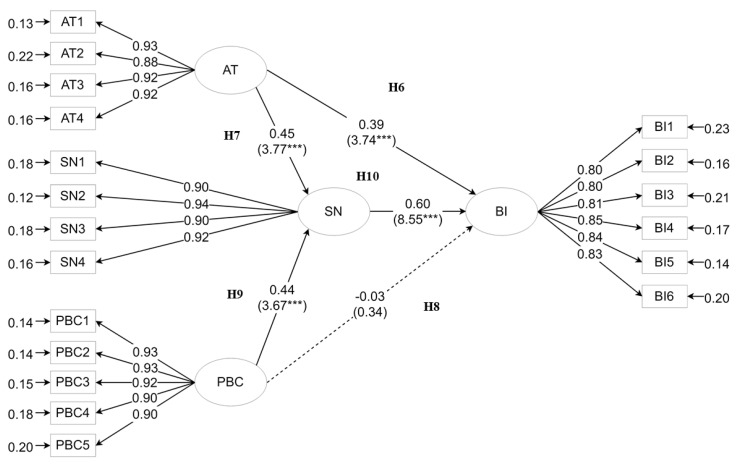
Path diagram of attitude (AT), perceived behavioral control (PBC), subjective norms (SN), and anti-PM2.5 behavioral intentions (BI) of parents from the rural areas in Beijing.

On the other hand, for rural parents, the CFI and NNFI values were 0.968 and 0.962, respectively (>0.9: good fit); the model GFI value was 0.877 (0.8–0.9: acceptable fit); the RMSEA was 0.076 (<0.080: acceptable fit); and the normed chi-squared value was 371.29 (>3). As shown in Figure 3, the path analysis highlighted a direct effect of attitude on anti-PM2.5 behavioral intentions (H6 was supported), but there was no evidence of a direct relationship between PBC and anti-PM2.5 behavioral intentions (H8 was rejected). However, an indirect relationship between PBC and anti-PM2.5 behavioral intentions could be developed through subjective norms (H10 was supported). Results also revealed the influence of attitude and PBC on subjective norms, and thus H7 and H9 were both supported.

## 4. Discussion and Implications

Based on the earlier mentioned TPB framework, this study conducted a survey to gain further insights on how the attitude, subjective norms, and PBC of parents of young children from urban and rural areas in Beijing could potentially affect their anti-PM2.5 behavioral intentions. Most studies have examined the influences of attitude, subjective norms, and PBC on behavioral intentions separately [42,54], but they have rarely considered the relationship among attitude, subjective norms, and PBC, and the effect of this relationship on behavioral intentions and their specific paths. This study aimed to fill this gap and further refine the TPB framework to determine its impact on anti-PM2.5 behavioral intentions. Findings revealed the support and accepted eight (i.e., H2, H3, H4, H5, H6, H7, H9, and H10) of the 10 hypotheses, indicating positive direct relationships.

### 4.1. Attitude

According to the TPB, attitude refers to the degree of a person’s support for a particular behavior [31]. Results showed that urban and rural parents in Beijing differed in their attitude towards anti-PM2.5 behavior intentions. For urban parents, attitude was found to have no direct impact on their anti-PM2.5 behavior intentions, but instead a moderate level of indirect influence was established through subjective norms. This could be explained with the fact that PM2.5 was attributed to manmade factors (such as transportation and coal burning) within the urban areas in Beijing. Furthermore, urban areas are situated further away from industrial areas, and thus urban parents were less concerned about the environmental pollution caused by economic and industrial developments and their associated environmental damage. Instead, they may be more concerned with issues closely related to their personal interests (e.g., inconvenience caused by transportation and heating issues) and to a lesser extent, about protection against PM2.5.

In contrast, the attitude of rural parents had a direct positive influence on their anti-PM2.5 behavior intentions, and this aligned with other empirical studies that agreed with this outcome [26,29,41,42,43]. For example, the main sources of PM2.5 in rural areas were external, for example industrial pollutants (e.g., from metallurgy processes and sulfates), with concerns from rural parents regarding the effects of smog and economic and industrial development-related environmental pollution on child health since these environmental problems were evident close to home [26].

### 4.2. Perceived Behavioral Control (PBC)

PBC is defined as the difficulty in consciously performing a particular behavior [31]; it is related to self-efficacy and control ability and is largely dependent on the costs and benefits in the process of performing the behavior, including economic cost, effort, and time [29]. The results of this study showed that the PBC of urban parents had a direct influence on their anti-PM2.5 behavioral intentions, in accordance with several previous studies [29,43,54]. Furthermore, the influence of PBC on subjective norms also revealed positive impact on anti-PM2.5 behavioral intentions. This could be explained by their higher levels of education and income, and greater access to other readily available resources that could contribute to behavioral intentions. For example, urban parents can install air purification equipment when they encounter smog pollution-related problems.

On the other hand, the PBC of rural parents had no direct influence on their anti-PM2.5 behavioral intentions, but instead established an indirect influence on their anti-PM2.5 behavioral intentions through their subjective norms. This contrasted with the results of previous studies and could be related to the lower levels of education and income of the rural parents, which had strong correlation with PBC [29].

Results showed that influencing rural parents’ anti-PM2.5 behavioral intentions through an indirect effect of subjective norms was crucial. In this process, the government could play an important role by establishing and improving infrastructure and formulating preferential policies to enhance the PBC of rural families [29]. This could include the provision of additional incentives and subsidy policies for rural areas, helping residents to purchase energy-efficient products and surgical masks, installing air purification equipment, improving air quality warning systems, and taking other relevant time- or cost-intensive protective actions against PM2.5. Moreover, local governments should also focus on educating parents, particularly those of young children, regarding protection against PM2.5, thus enhancing their PBC.

### 4.3. Subjective Norms

Subjective norms refer to social pressures exerted by the important others on a person when deciding to perform a certain behavior [31]. The results of this study indicated a direct positive path relationship existed between subjective norms and anti-PM2.5 behavioral intentions in both the urban and rural parents. Subjective norms were not only the strongest direct predictors of anti-PM2.5 behavioral intentions, but also played an important role to attitude and PBC as an indirect path to influencing anti-PM2.5 behavioral intentions. The results were consistent with the findings of previous studies that confirmed a significant correlation between subjective norms, PBC, and attitude [30,41,42,44,45,46], with attitude directly affecting subjective norms and indirectly affecting anti-PM2.5 behavioral intentions [45,47]. Subjective norms also had an indirect impact on anti-PM2.5 behavioral intentions through PBC and attitude [43] encouraging certain behaviors [48,49,51,52,53]. This was particularly relevant in the Asian society context, where norms were more greatly valued. Subjective norms constitute a type of social norms created by surrounding groups, social influences, and social factors shaping people’s behaviors [60]. In China, individuals are usually willing to fulfill the expectations of other individuals or organizations, potentially because of collectivism, which is part of the country’s longstanding traditional culture. Chinese residents are more concerned about the views of other individuals or organizations when they decide whether to take certain actions [29]. For example, these expectations of important others or organizations regarding protection against PM2.5 could potentially affect the parents’ anti-PM2.5 behavioral intentions. Therefore, the inclusion of related subjective norms to the protection against PM2.5 to relevant education might be essential for enhancing parents’ anti-PM2.5 behavioral intentions.

The level of education might be related to personal attitude, which could further affect subjective norms [61]. Subjective norms, PBC, and attitude interact with anti-PM2.5 behavioral intentions [53,55]. Therefore, inviting important celebrities or institutions to participate in activities for mass media and public service advertisements that promote protection against PM2.5 (e.g., demonstrating appropriate mask use, energy-saving product use, and air quality warning interpretation) could be helpful to improve the parents’ subjective norms, protective attitude, PBC, and anti-PM2.5 behavioral intentions.

### 4.4. Regulatory and Health Implications

The findings of this study provide further insights into regulatory and health education for urban and rural parents from Beijing regarding protection against PM2.5. The government should continue promoting protection against serious smog pollution and PM2.5 through various channels, such as school education, community mass media, and public service advertisements, and celebrity endorsements. In addition, the government should also encourage the strengthening of school environmental protection and health education, the use of home-school cooperation, especially for the education of young children’s parents, so as to improve PM2.5 protection in terms of PBC.

For rural parents, the government should establish and improve the infrastructure platform and formulate preferential policies such as the provision of additional incentives and subsidies for rural areas to help residents purchase energy-saving products, and protective masks to enhance PBC with respect to rural parents’ PM2.5 protection. As for urban parents who have higher educational levels and PBC advantages, the government should encourage them to purchase and utilize more environmentally friendly PM2.5 emission reduction and health protection products/equipment that could be beneficial to their children in the school environment and health education, so as to enhance their attitude toward PM2.5 protection.

## 5. Conclusions, Limitations and Future Research

In conclusion, this study explored the determinants of anti-PM2.5 behavioral intentions of urban and rural parents of young children in Beijing and reported that subjective norms were most influential for predicting anti-PM2.5 behavioral intentions for both rural and urban parents. Findings also revealed that the strengths and paths of PBC and attitude differed between urban and rural parents. For urban parents, PBC had a direct impact on their anti-PM2.5 behavior intentions, and their attitude also had an indirect influence on behavioral intentions through subjective norms. On the other hand, rural parents’ attitudes had direct/indirect effects, and PBC had an indirect effect on anti-PM2.5 behavioral intentions through subjective norms.

Beijing has a large metropolitan population in both urban and rural settings. However, the findings of this study are only applicable to Beijing and cannot be generalized to the population at large in China. A more representative sampling population should be sought and tested in order to generalize the findings. Further studies from other countries would be needed to provide international comparisons and ascertain similarities or differences. In addition, future studies may also seek to explore the impacts of protection against PM2.5, environmental sensitivity, social norms, and other factors on related behavioral paths.

## Figures and Tables

**Table 1:** Descriptive statistics and difference analyses related to demographic issues of attitude, subjective norms, perceived behavioral control, and behavioral intentions.

Variables	Frequency	Percent (%)	Attitude				Subjective Norms				Perceived Behavioral Control				Behavioral Intentions			
			Mean	SD	*t*	*p*	Mean	SD	*t*	*p*	Mean	SD	*t*	*p*	Mean	SD	*t*	*p*
**Region**																		
Urban Beijing	1012	79.2	4.21	0.99	4.727	0.000	4.24	0.85	5.529	0.000	4.31	0.94	6.155	0.000	4.31	0.84	6.838	0.000
Rural Beijing	265	20.8	3.88	1.12			3.89	1.08			3.90	1.12			3.89	1.06		
Gender																		
Female	879	69.4	4.23	0.99	4.113	0.000	4.22	0.88	3.293	0.001	4.31	0.96	4.088	0.000	4.31	0.87	4.874	0.000
Male	387	30.6	3.97	1.06			4.04	0.96			4.06	1.03			4.04	0.96		
**Urban Beijing** Gender																		
Female	709	70.8	4.29	0.95	3.422	0.001	4.28	0.82	2.268	0.024	4.38	0.90	3.257	0.001	4.38	0.80	3.716	0.000
Male	293	29.2	4.05	1.04			4.14	0.90			4.17	0.98			4.16	0.89		
**Rural Beijing** Gender																		
Female	170	64.4	3.98	1.12	1.833	0.068	3.99	1.07	2.075	0.039	4.01	1.12	2.162	0.032	4.02	1.05	2.627	0.009
Male	94	35.6	3.71	1.11			3.70	1.07			3.70	1.10			3.66	1.06		

**Table 2:** Cross-analysis of educational backgrounds between parents from urban and rural areas in Beijing.

Place of Residence	Educational Background			Total
	High School Graduates and Below	University or College Graduates	Master’s Studies and Above	
Urban areas	108 (10.67%)	740 (73.12%)	164 (16.21%)	1012
Rural areas	65 (24.53%)	180 (67.92%)	20 (7.55%)	265
Total	173 (13.55%)	920 (72.04%)	184 (14.41%)	1277

**Table 3:** Difference analysis of educational backgrounds when comparing parents from urban and rural areas in Beijing.

	Frequency	Percent (%)	Mean	SD	*t*	*p*
Urban areas	1012	79.2	2.06	0.52	8.237	0.000
Rural areas	265	20.8	1.83	5.41		

**Table 4:** Cross-analysis of incomes between parents from urban and rural areas in Beijing. 1 Chinese Yuan (RenMinBi, RMB) = 0.144 U.S. Dollars.

Place of Residence	Income (RMB)					Total
	Less Than 100,000	110,000–200,000	210,000–300,000	310,000–400,000	Over 410,000	
Urban areas	268 (26.48%)	368 (36.36%)	232 (22.92%)	77 (7.61%)	67 (6.62%)	1012
Rural areas	107 (40.38%)	80 (30.19%)	54 (20.38%)	18 (6.79%)	6 (2.26%)	265
Total	375 (29.37%)	448 (35.08%)	286 (22.40%)	95 (7.44%)	73 (5.72%)	1277

**Table 5:** Difference analysis for incomes between parents from urban and rural areas in Beijing.

	Frequency	Percent (%)	Mean	SD	*t*	*p*
Urban areas	1012	79.2	2.32	1.14	4.032	0.000
Rural areas	265	20.8	2.00	1.04		

**Table 6:** Descriptive statistics for attitude (AT)-related items.

Attitude (AT)	Mean	SD
AT1. I care about whether smog affects the health of the people.	4.16	1.08
AT2. I care about some problems of domestic industrial pollution.	4.16	1.08
AT3. I care about the environmental problems resulting from economic development.	4.11	1.10
AT4. Smog pollution is the result of human activities destroying the environment.	4.15	1.09
Overall attitude	4.16	1.02

**Table 7:** Descriptive statistics for subjective norm (SN)-related items.

Subjective Norms (SN)	Mean	SD
SN1. People important to me hope that I choose environmental-friendly products.	4.14	0.98
SN2. People important to me hope that I walk, ride a bicycle or use modes of public transport.	4.16	0.97
SN3. People important to me hope that I wear a surgical mask and get my children to wear surgical masks when there is smog.	4.21	0.98
SN4. People important to me hope that I participate in environmental activities to improve smog pollution.	4.16	0.98
Overall subjective norms	4.17	0.91

**Table 8:** Descriptive statistics for perceived behavioral control (PBC)-related items.

Perceived Behavioral Control (PBC)	Mean	SD
PBC1. We can carry out protective work against PM2.5.	4.22	1.05
PBC2. I can guide my children to protect themselves against PM2.5.	4.20	1.06
PBC3. I can give environmental education to my children before they start school (at the kindergarten stage).	4.23	1.04
PBC4. I can install air purification equipment with good purification and ventilation functions.	4.25	1.05
PBC5. I pay attention to poor air quality and respond by taking measures.	4.24	1.04
Overall perceived behavioral control	4.22	0.99

**Table 9:** Descriptive statistics for anti-PM2.5 behavioral intentions (BI)-related items. PM2.5: fine particulate matter with an aerodynamic diameter of ≤2.5 μm.

Behavioral Intentions (BI)	Mean	SD
BI1. I use fuel that has less impact on the environment even if it costs much more.	4.10	1.01
BI2. I participate in activities related to environmental protection.	4.19	0.97
BI3. I advise others against polluting the environment.	4.20	0.97
BI4. When the smog is serious, I will get the children to wear surgical masks fitting their faces tightly.	4.29	1.00
BI5. When the smog reaches the warning level, I get the children to stay indoors.	4.30	0.99
BI6. I pay attention to the Air Quality Index (AQI) every day to remind the children to pay attention to protect themselves against smog.	4.27	1.01
Overall behavioral intentions	4.22	0.91

**Table 10:** Pearson’s correlation matrix (mean).

	Attitude	Subjective Norms	Perceived Behavioral Control	Behavioral Intentions
Attitude	1.000			
Subjective norms	0.739	1.000		
Perceived behavioral control	0.844	0.804	1.000	
Behavioral intentions	0.751	0.888	0.815	1.000

All correlations are significant, *p* < 0.001, two-tailed test.
